# Integrating palliative care with intensive care for critically ill patients with lung cancer

**DOI:** 10.1186/2110-5820-2-3

**Published:** 2012-02-16

**Authors:** Elizabeth B Gay, Stefanie P Weiss, Judith E Nelson

**Affiliations:** 1 Department of Pulmonary and Critical Care Medicine, University of Virginia Health Systems, Charlottesville, VA; 2 Department of Medicine, Division of Pulmonary, Critical Care, and Sleep Medicine, Mount Sinai School of Medicine, New York, NY

## Abstract

With newer information indicating more favorable outcomes of intensive care therapy for lung cancer patients, intensivists increasingly are willing to initiate an aggressive trial of this therapy. Concerns remain, however, that the experience of the intensive care unit for patients with lung cancer and their families often may be distressing. Regardless of prognosis, all patients with critical illness should receive high-quality palliative care, including symptom control, communication about appropriate care goals, and support for both patient and family throughout the illness trajectory. In this article, we suggest strategies for integrating palliative care with intensive care for critically ill lung cancer patients. We address assessment and management of symptoms, knowledge and skill needed for effective communication, and interdisciplinary collaboration for patient and family support. We review the role of expert consultants in providing palliative care in the intensive care unit, while highlighting the responsibility of all critical care clinicians to address basic palliative care needs of patients and their families.

## Introduction

Appropriate use of intensive care therapies for patients with lung cancer is a topic of continuing research and clinical interest. There has been a focus on issues related to intensive care unit (ICU) triage decision-making, such as whether expectations for clinically meaningful outcomes justify the burdens and costs of critical care treatment for lung cancer patients, and whether predictors at the time of ICU admission or during a trial of intensive care can help to refine our evaluation of potential risks and benefits [1-6]. As new data emerge from studies of lung cancer screening, staging, drug development, and molecular diagnostics, prospects for reducing mortality from this disease appear to be growing [7], whereas advances in critical care are concomitantly improving outcomes for ICU patients. These data, including specific information about outcomes of intensive care therapy for patients with lung cancer, have driven an increasing receptivity on the part of intensivists to initiate this therapy and to pursue it aggressively, at least for a time-limited trial [1,3,4,6,8]. Concerns remain, however, that the experience of the ICU for lung cancer patients and families, and their longer-term outcomes, often may be unfavorable [9].

In this article, we start from the assumption that lung cancer patients will continue to be cared for in ICUs, likely with increasing frequency, in the foreseeable future [10]. We also accept the premise that all patients with critical illness, regardless of prognosis, should receive high-quality palliative care comprising the following core elements: alleviation of symptom distress; communication about care goals; alignment of treatment with patients' values and preferences; transitional planning; and support for both patient and family throughout the illness trajectory [11-14]. For our framework, we favor a concurrent model in which palliative care and intensive care are provided together as synergistic approaches, rather than a sequential model that defers palliative care until intensive care fails [15]. Accumulating data suggest that early and ongoing integration of palliative care can enhance the effectiveness of disease-directed treatment for patients with serious illness, including lung cancer [16]. Based on existing evidence, we suggest strategies for integrating palliative care with intensive care for critically ill lung cancer patients and their families.

### Symptom distress: assessment and management

Many clinicians still understand "comfort care" as a euphemistic code for limitation of intensive care and disease-directed therapies. Seen as an end-of-life approach exclusively, "comfort care only" begins when such therapies are withheld or withdrawn from patients who are expected to die imminently. Yet, this narrow view of the role of symptom control for critically ill patients lacks empirical support and ignores evidence that alleviation of physical and psychological distress can mitigate maladaptive physiologic responses to such distress while facilitating stability and recovery [16-20]. Systematic assessment and effective management of symptoms are key components of comprehensive care for lung cancer and other patients with critical illness, including those receiving aggressive treatments to extend life.

Symptom burden is significant across the trajectory of lung cancer, from the time of diagnosis through advanced stages of illness and even after survival of treatment with curative intent. Patients with lung cancer experience a broad range of symptoms [21-27] that are associated not only with patient distress and impairments of function and quality of life [24,25,27] but with poorer survival [18,19,28,29]. A recent study of 276 patients with lung cancer who were interviewed within the first months after diagnosis, among whom a majority had stage I or II disease, found that more than 1 in 5 patients rated pain within the prior week at the highest levels on the scale ("quite a bit" and "very much") [30]. Patients with cancer often have multiple different types of pain: including somatic pain from involvement by the primary tumor or metastases of pleura, ribs, chest wall, spine, or other bones; neuropathic pain from chest or brachial plexus nerve involvement; visceral pain from tumor invasion of various organs; and pain associated with anticancer therapy, including surgery [31]. Among a large cohort of hospitalized patients with advanced lung cancer, more than 25% reported severe pain and/or dyspnea; of those dying within 2 months, 35% had severe pain and 46% had severe dyspnea at least half the time in the hospital [32]. Psychological distress is more prevalent in lung cancer patients than those with other solid tumors [33], affecting even those receiving successful disease-directed treatment as well as others [34-37] and interacting with and exacerbating other symptoms, such as pain [38].

Limited evidence describes the symptom experience of critically ill patients with malignancy. In a prospective study of 100 medical ICU patients with a present or past diagnosis of cancer and a hospital mortality rate of 56%, 50 were able to provide self-reports of symptoms assessed by the Edmonton Symptom Assessment Scale [39]. Between 55% and 75% of responders rated pain, discomfort, anxiety, sleep disturbance, or unsatisfied hunger or thirst as moderate or severe. Dyspnea and depression at these levels were reported by approximately 33% and 40% of responders. A retrospective study reviewing the experience of 88 cancer patients seen in consultation in the ICU by the hospital's palliative care team found that eight of ten symptoms in the Edmonton Symptom Assessment Scale were reported by 60% or more of the patients; pain and dyspnea were reported by more than three-quarters of patients [40]. Another study evaluated presence, intensity, and distress of multiple symptoms through more than 400 interviews with 171 critically ill patients deemed to be at high risk of dying (one-third died), of whom 22% had cancer [41]. Pain and shortness of breath were reported in 40% and 44% of these assessments, respectively; between 50% and 75% of assessments revealed anxiety, thirst, and fatigue, and many patients also reported other physical and psychological symptoms. In a prospective, observational study conducted in 44 ICUs in France, assessing almost 1,400 patients, including 16% with active cancer and 6% receiving recent chemotherapy for cancer, more than half of patients providing ratings on a visual analog or verbal descriptive scale reported pain at substantial levels [42]. As intensivists reduce the use of sedating medications to promote shorter duration of mechanical ventilation and earlier patient mobilization, an even higher prevalence and intensity of physical and emotional symptoms during acute critical illness may be unmasked.

Systematic symptom assessment, as recommended by major societies representing critical care professionals [43] and included within standards of The Joint Commission [44], is the cornerstone of effective symptom control. A before-after study in a medical-surgical ICU in France found that an intervention consisting of regular, frequent, and standardized assessments by nurses with required reporting of pain or agitation above specified threshold levels was associated with significant decreases in the incidence of pain and agitation, closer titration of analgesic and psychoactive drugs, shorter duration of mechanical ventilation, and fewer nosocomial infections [45]. That is, pain control and other outcomes improved with assessment, in the absence of a protocol mandating treatment. For self-reports of symptoms, which remain the "gold standard" of assessment, palliative care researchers have developed simple, practical symptom measurement tools that avoid undue burden and provide sufficient information for clinical management. Examples that measure a diverse group of symptoms included the Condensed Form of the Memorial Symptom Assessment Scale [46], which can be modified for use with critically ill patients [47], and the Edmonton Symptom Assessment Scale [48]. Abbreviated instruments also are available to evaluate specific symptoms, such as pain [49], dyspnea [50], and depression [51]. Several tools are available to assess symptoms, such as pain and dyspnea, with patients who cannot self-report because of impaired cognition and/or inability to communicate, which often occurs in the ICU: these include the Behavioral Pain Scale [52], Critical Care Pain Observation Tool [53], Assume Pain Present approach [54], and Respiratory Distress Observation Scale [55]. Lung cancer-specific assessment tools include a composite of the 3-symptom subscales (physical well-being, emotional well-being, and lung cancer-specific concerns-totaling 20 items) of the Functional Assessment of Cancer Therapy-Lung (FACT-L) [56], and the 13-item lung-cancer-specific module supplementing the European Organization for Research and Treatment of Cancer-Quality of Life Questionnaire (EORTC-QLQ-LC13) [57]. These tools were designed and validated as research instruments, however, and would be difficult to use in clinical care of critically ill patients.

The National Comprehensive Cancer Network publishes and annually updates robust clinical practice guidelines for treatment of cancer pain [58]. Although other expert recommendations specifically address management of pain and other symptoms in patients with lung cancer [38,59], these are not readily applied in the context of critical illness. On the other hand, general recommendations for analgesic management of critically ill patients [43] need further support from rigorous, large-scale trials and may not account for specific needs of patients with lung cancer and other malignancies, whose acute distress often is superimposed on chronic pain and other symptoms and attributable both to the underlying disease and to anticancer therapies. Of particular concern in ICUs is the practice of undertreating pain and other symptoms in the face of instability resulting from the critical illness, sometimes accepting a patient's distress rather than increasing vasopressor or ventilator support to address underlying issues, such as sepsis-induced hypotension or encephalopathy.

Given the range of barriers to effective management of pain in the ICU setting, structured approaches may improve ICU pain treatment [60]. Suggestions include involvement of an interdisciplinary team to develop an improvement process, implementation of tools, such as clinical paths and checklists, incorporation of analgesic management approaches within electronic health records and computerized provider order entry systems, and referrals for specialist input on particularly challenging cases [60], Clinicians on the ICU team should have basic knowledge of equianalgesic opioid dosing; this information is easily incorporated in computer-based ordering systems and/or on pocket cards for clinicians [61]. In addition, recognition and proactive management of common side effects of opioid treatment are important competencies for critical care practice. Therapeutic strategies can refer to evidence-based guidelines for palliative care of cancer patients [58], although these do not speak directly to care in ICU settings. For specific issues that arise frequently in the ICU, such as the increased risk in renal failure of neurotoxicity from accumulation of active metabolites of morphine [62,63], targeted education and system supports (e.g., automatic pharmacy warnings) are appropriate.

For dyspnea, which is reported by patients receiving mechanical ventilation [64] as well as others with critical illness, opioids remain the most effective pharmacologic treatment and usually are therapeutic at much lower doses than are needed for pain [65,66]. Other interventions for dyspnea in lung cancer patients are reviewed elsewhere [38,67,68]. However, empirical data on management of dyspnea in the ICU are scant. The role of palliative noninvasive ventilation for life support and for relief of dyspnea in patients who are not receiving endotracheal mechanical ventilation is still being defined [69,70]. As discussed more fully below, specialists in palliative care, where available, can help the ICU team with the complex challenges that may arise when providing life-prolonging measures along with symptom control.

### Communication with lung cancer patients and their families during critical illness

Recent data document that across all stages of the disease, many lung cancer patients have not communicated with clinicians primarily responsible for their oncologic care about key issues related to treatment decision-making, including preferences for use of intensive care therapies [30,71]. For example, a recent, prospective study used a standardized questionnaire to obtain patient ratings of the extent of communication with lung cancer physicians about 11 important topics, including prognosis, potential complications of therapy, care goals, life support preferences, proxy appointment, living will preparation, symptoms, and other concerns [30]. Among 276 patients at 4 major medical centers in New York City, 1 in 5 reported that their physicians communicated "not at all" or "a little bit" on all topics in the questionnaire; 40% gave these low ratings to communication about the chances of curing the lung cancer; and more than 80% reported low levels of communication regarding resuscitation/life-sustaining treatment and preparation of an advance directive. However, 40% of the study patients who had a preference with respect to resuscitation stated that they would not want resuscitation to be attempted in the event of an arrest, and 60% would not want mechanical ventilation for respiratory failure. Overall, 52% of patients reported that communication with physicians was inadequate, a proportion that was similar across patients of all pathologic stages. In the U.S. Cancer Care Outcomes Research and Surveillance (CanCORS) study, barely 50% of more than 1,500 patients with stage IV lung cancer reported discussing hospice with a care provider, yet more than 70% of those patients died within 6 months (the time period for hospice eligibility) of the study interview [72]. The majority of more than 4,000 physicians caring for CanCORS patients indicated that, despite national guidelines recommending discussion of prognosis, resuscitation status, hospice, and preferred site of death with patients expected to die within a year, they would not initiate such a discussion even if death were expected within 6 months. Rather, they would postpone discussions until the patient became symptomatic or failed disease-directed treatment and might never conduct these discussions unless initiated by the patient or family [71].

Physicians' reluctance to discuss prognosis, preferences for intensive care therapies, and issues related to end-of-life care reflects in part concerns that such communication would extinguish hope or worsen emotional distress [73]. Research shows, however, that cancer patients and their families generally prefer to be informed even if the disease is incurable and most patients favor early disclosure of poor prospects [74,75]. Similarly, most surrogates of patients with critical illness understand and accept that physicians cannot prognosticate with certainty but still prefer to discuss expectations for outcomes even if they are uncertain or unfavorable [76]. Most of these surrogates believe that physicians should not withhold information about prognosis as a way to preserve hope and consider prognostic information to be essential for emotional and practical preparation [76]. In a U.S. multisite study of patients with advanced cancer and their informal caregivers, rates of major depressive disorder were not higher among patients who had discussed preferences for care at the end of life with their physicians, but were higher among bereaved caregivers of patients who had not had such discussions [77]. In fact, compared with those receiving standard oncologic care alone, ambulatory outpatients with advanced lung cancer who also received consultation from palliative care specialists addressing understanding of the illness, treatment decision-making, and development of care plans had better mood and quality of life along with improved understanding of their prognosis [16]. On the other hand, cancer patients who overestimate their chances of survival are more inclined to choose treatments for which burdens outweigh potential benefits [78], less likely to discuss care preferences with their surrogate decision-makers [79], and less likely to obtain information that might improve the quality of their experience at the end of life [72].

Successful communication about care goals requires both knowledge and skill on the part of clinicians, who should review recent data on outcomes of ICU treatment for critically ill patients with lung cancer and master evidence-based approaches for communicating with such patients and their families. Whereas older studies suggested that potential benefits of intensive care for patients with lung cancer rarely justified burdens and costs, short-term survival after treatment for critical illness seems to be improving for patients with lung cancer as for those with other malignancies. ICU and hospital survival rates of 78% and 60%, respectively, were documented in a retrospective study of 139 lung cancer patients admitted between 1998 and 2005 to the medical ICU of a university-affiliated medical center in the United States [1]. For patients who required mechanical ventilation, who comprised half of the cohort, ICU survival was 62% and 47% were alive at hospital discharge [1]. In hospitals in France and Brazil that specialize in cancer care, hospital survival among 143 patients with lung cancer in two medical ICUs was approximately 40% overall and 30% for 100 patients in the study group who were mechanically ventilated [4]. A separate, retrospective study conducted in a lung cancer referral center in France evaluated 6-month survival for 105 consecutive lung cancer patients, including more than 80% with advanced non-small cell or disseminated small cell cancer but excluding those who had undergone surgical resection of lung cancer during the same hospital stay [5]. Although 6-month survival was less than 30%, two-thirds of the ICU survivors were able to receive anticancer treatments after discharge. A retrospective study of 103 patients with unresectable lung cancer in three French ICUs found a 3-month survival rate of 37%, and 12% of patients were alive at 1 year [3]. In studies of heterogeneous cohorts of cancer patients and of lung cancer patients specifically, factors associated with poorer outcome have included poor performance status before ICU admission [2,3,5], need for vasopressor support [6,80,81], cancer disease progression [2,5], use of invasive mechanical ventilation [3,5,6], and persistent or worsening organ dysfunction after several days of ICU treatment [3]. Such data can be helpful to clinicians to prepare for discussions with lung cancer patients and families about achievable goals of intensive care.

Other data are relevant for informing discussions of resuscitation status, because risks and benefits of cardiopulmonary resuscitation (CPR) in case of an arrest are distinct from those of a trial of intensive care. A national study of CPR outcomes in critically ill patients in the United States included patients with hematologic or metastatic cancer, who comprised 11% of the cohort of almost 50,000 patients [82]. For the entire cohort, survival to hospital discharge was less than 16% and the proportion discharged to home was 8.5%. Among those who required vasopressors or mechanical ventilation, outcomes were worse [82]. Fewer than 4% of patients who received vasopressors and CPR were discharged to home, and still fewer went home with a favorable neurological outcome [82]. Mechanical ventilation was associated with 40% lower odds of hospital survival after CPR [82]. Although the presence of malignancy, by itself, has not been identified as an independent predictor of hospital survival or functional outcome after CPR, the prognosis of the underlying cancer along with the nature and evolution of the critical illness provide a context for discussing resuscitation status. Data suggest that in practice, resuscitation status has not been clarified before ICU admission for most lung cancer patients [30,40,83] (who then are "full code" by default) but that a "Do Not Resuscitate" directive is entered before death for approximately half of these patients [83].

Qualitative investigations of clinician communication in the ICU and in outpatient cancer care settings suggest specific skills and approaches to improve the effectiveness of this communication. Listening is a key skill in which few physicians have received training or achieved mastery. Audio recordings of ICU family conferences reveal that physicians generally dominate these discussions, leaving little opportunity for active participation by the family, investigation of the patient's values and preferences, or attention to family concerns [84]. Yet, family satisfaction with these conferences is associated with the proportion of time in which the family is speaking relative to the time in which clinicians are speaking to them [85]. To encourage comments and questions from family members, the "Ask-Tell-Ask" approach has been described [86]. The clinician begins the discussion by asking family members to report their understanding of a situation (e.g., the patient's condition and prognosis), including what they have been told by other clinicians, and by asking permission to continue with the discussion. The clinician then provides a succinct update of the situation in layperson's terms, showing sensitivity to differences in health literacy and cultural background. The family is then asked again to summarize the discussion, comment, ask questions, and share concerns. A rigorous process, including literature review, surveys of critical care clinicians, and structured interviews of clinicians and families, identified questions of importance to family members in the ICU [87], and these questions could be specifically explored by clinicians if not brought forward by families themselves.

Evidence also indicates the importance of acknowledging and addressing emotions during clinician communication with seriously ill patients and their families, while documenting that physicians often fail to demonstrate this skill [84,88,89]. Strong emotions can overwhelm the ability to absorb and integrate information needed by the patient and family to make rational decisions about treatment or establish appropriate care goals. Thus, clinicians should incorporate explicit expressions of empathy, which help to control emotional fluctuations and distractions, and thereby enhance cognitive processing of important clinical information. The acronym "N.U.R.S.E." abbreviates statements that communicate empathy explicitly: **N**ame the emotion to make clear that it is recognized; express **U**nderstanding in an open and compassionate way; show **R**espect for the person experiencing the emotion; communicate **S**upport; and **E**xplore the emotional experience of the other person in greater depth [89]. Examples of empathic statements in these domains are available as guides for clinicians [89,90].

For communication addressing limitation of ICU therapies, data show that families are more satisfied when clinicians provide assurance that the patient will not be abandoned before death and will not suffer, and support the family's decision whether it is to forgo or to continue therapy [91]. Expert recommendations have been provided for communicating with patients and family members who choose to continue life-supporting treatments in the hope that a miracle will occur [92], and those who demand "everything," including treatments deemed nonbeneficial by clinicians.

### Support of the ICU family

Evidence of the emotional, physical, and practical burdens for families of seriously ill patients, including those who receive intensive care treatment, continues to accumulate. Depression and anxiety are prevalent among ICU families, as are acute and posttraumatic stress symptoms that can persist and even increase over time [93,94]. Responsibility for making life-death decisions as surrogates weighs heavily [76,95]. For families whose loved ones die in the ICU or soon after, which is still common for critically ill patients with lung cancer, grief often is complicated [93,96]. In follow-up interviews with bereaved family members of patients who died in the ICU, one-third met DSM-IV criteria for at least one psychiatric disorder (major depression, generalized anxiety, panic, or complicated grief) [96]. Many families of ICU survivors, especially those facing an ongoing serious illness, such as lung cancer, remain burdened in multiple ways and over long periods. Qualitative research captures themes of regret, exhaustion, isolation, and hopelessness as expressed in caregivers' own words [97]. Personal and professional lives may be disrupted [98]. Among families of patients who received mechanical ventilation for as little as 3 days, post-ICU decrements in physical health and health-related quality of life are common, and the experience of role overload intensifies over time [98,99]. Informal caregivers who feel strained by this role are known to be at higher risk of mortality [100]. Thus, the term "post-intensive care syndrome," which was recently recommended by a consensus of experts to describe "new or worsening impairments in physical, cognitive, or mental health status arising after critical illness and persisting beyond acute care hospitalization," is applicable to family members as well as to surviving ICU patients [101]. Support for family members enables them to function more effectively as caregivers and surrogate decision-makers, thereby also benefiting patients and clinicians. Both patients and family members identify family care as a core domain of high-quality palliative care in the ICU [14].

Randomized, controlled research shows that proactive, sensitive, and structured communication by clinicians improves psychological well-being of ICU families as well as their satisfaction. For families facing the imminent death of a loved one in the ICU, an intervention, including a protocol-driven discussion with clinicians and a brochure addressing bereavement, was associated with a lower prevalence and severity of posttraumatic stress disorder, anxiety, and depression at 3-month follow-up [102]. A variety of other printed informational aids have been developed to support and supplement clinician counseling of ICU families [103,104], including a Family Meeting Brochure intended to guide families in preparing for a face-to-face discussion about the patient's condition, prognosis, and appropriate goals of care [103,105]. A leaflet providing general information about the ICU improved family understanding of acute critical illness and treatment and increased family satisfaction [106]. A brochure developed and validated to provide information and prompt further discussion about protracted critical illness includes the potential long-term impact on the patient's cognition and function and burdens faced by families [104]. As research continues to clarify outcomes for patients with lung cancer and other malignancies, who undergo treatment for critical illness, this knowledge might be incorporated in printed aids and other educational resources to help families anticipate and manage challenges they face.

Patients as well as families place high value on family access and proximity to the patient [14]. Patients report their awareness of family presence and the comfort and strength it gives them. Families identify many important functions they serve, including their role as advocates, monitors, protectors, interpreters, caregivers, and decision-makers [14,107]. In addition, families perceive their presence as essential for optimal communication with the ICU team and for alleviation of their own distress [14]. These data provide support for a liberal approach to family visiting [108-110]. Although clinicians might prefer that families leave the bedside during daily ICU rounds, some have adapted their practice to allow families not only to remain but to participate [109], an approach recommended by a consensus of expert opinion [109]. If skillfully led and coordinated, such rounds can help to address family needs for information and support without distracting from the care of critically ill patients, education of intensive care trainees, or other exchanges within the ICU team.

Contributions from multiple members of the interdisciplinary ICU team enhance support for families [14]. The role of the nurse in optimally integrating palliative care with intensive care has been emphasized [111]. Among other essential functions, nurses can help to report patient symptoms and family concerns and to communicate about achievable goals of treatment that are consistent with patient preferences [111]. Other disciplines, such as pastoral care and social work, also provide perspectives and services that complement the work of the medical team. More research needs to focus on models for coordinating interdisciplinary input to strengthen family support within a comprehensive framework of care for critically ill patients with lung cancer or other conditions during the ICU stay and beyond.

### Role of expert consultants in providing palliative care in the ICU

Following a decade of exponential growth, palliative care specialists are now available at 85% of acute care hospitals with 300 or more beds and half of hospitals with at least 50 beds in the United States [112,113]. In 2006, the subspecialty of palliative medicine achieved recognition by the American Board of Medical Specialties, receiving broad cosponsorship from an unprecedented number of specialty boards. The field of palliative care also is growing in other countries. When available, specialists in palliative care can provide expert input on management of symptoms, discussions of care goals, and family support for critically ill patients with lung cancer and others with serious and complex illness. They can assist with transitional planning and provide continuity across multiple venues of care. Some ICUs have adopted a "consultative model," giving palliative care specialists a major role, particularly in the care of patients at highest risk for poor outcomes [114]. Consultations may be "triggered" by specified criteria, which could include a diagnosis of advanced cancer [115], or a palliative care clinician may join ICU rounds on a regular basis to help with timely identification of patients and families who could benefit from expert input [116,117]. In other ICUs, where an "integrative model" is used, the critical care team incorporates palliative care principles and interventions for all patients and families in the ICU [114]. A third model blends features of the other two, relying on the ICU team to meet basic palliative care needs of patients and families while engaging specialists for more complex or refractory problems [114].

Benefits of consultation by palliative care specialists include improved symptom control, clearer family understanding of diagnosis and prognosis, more efficient utilization of ICU resources, and enhanced patient and family satisfaction [16,115,118-120]. In a recent, randomized, controlled trial, early integration of palliative care with standard oncologic care for ambulatory patients with stage IV lung cancer was associated not only with better mood and quality of life but also with longer survival compared with standard oncologic care alone [16]. The National Comprehensive Cancer Network and American Society of Clinical Oncology support early integration of palliative care for all cancer patients [121,122]. The American College of Chest Physicians' Evidence-Based Clinical Practice Guidelines for Diagnosis and Management of Lung Cancer support integration of palliative care, including involvement of a palliative care consultation team, specifically extending its recommendations to include patients pursuing curative or life-prolonging therapies [123]. Utilization of palliative care consultative services in the care of ICU and other hospitalized patients with lung cancer has been described [40,124].

Even where they are available to provide consultative input, existing data indicate that palliative care specialists are underutilized for lung cancer patients. A recent survey of physicians caring for lung cancer patients at five separate medical centers with established, interdisciplinary palliative care teams found that among 155 (78%) responders, half reported referring < 25% of these patients for palliative care consultation [125]. In multiple regression analysis, physicians' belief that referral to a palliative care specialist would alarm patients and families was a significant predictor of low referral, whereas the belief that palliative care specialists spend more time discussing complex issues was a significant predictor of more frequent referral. In a study of palliative care consultations for lung cancer patients who received treatment in the ICU of a large comprehensive care center, among whom three-quarters had multiple comorbid conditions, the mean duration between ICU admission and referral to the palliative care service was 10 days [40]. More than 90% of these referrals were made by the primary oncology team, whereas less than 10% were from the ICU team. The vast majority of these patients were experiencing severe symptom distress and delirium, which improved after interventions by the palliative care team. In addition, although few (12%) patients had an advance directive before the consultation, and most (81%) had "full code" status at that time, decisions were made not to attempt resuscitation in the event of arrest for 70% of patients after discussions with the interdisciplinary palliative care team, which included a social worker, psychiatric nurse counselor, and pastoral care provider. More than 40% of the lung cancer patients seen in palliative care consultation were alive at hospital discharge. Such data support assiduous efforts to ensure timely access to specialist palliative care for a broader group of patients.

## Conclusions

The future is likely to bring an increasing number of critically ill patients with lung cancer to the ICU. Although specialists in palliative care will be increasingly available, attention to basic palliative care needs of patients and families remains an ongoing responsibility for intensive care clinicians. Critical care and palliative care are not mutually exclusive but, rather, mutually enhancing approaches to the care of ICU patients, including those with lung cancer. Through early and continuing integration of these approaches, intensivists can improve patient and family well-being while optimizing disease-directed and restorative treatments.

## Competing interests

The authors declare that they have no competing interests.

## Authors' contributions

EBG and JEN conducted the literature review. All authors contributed to drafting of the manuscript, and all have read and approved the final manuscript.
